# Long‐Term Effects of Left Bundle Branch Area Pacing Versus Traditional Right Ventricular Pacing on Atrial Fibrillation After Dual‐Chamber Pacemaker Implantation

**DOI:** 10.1002/clc.70116

**Published:** 2025-03-18

**Authors:** Jieruo Chen, Zefeng Wang, Fei Hang, Weiping Sun, Haiwei Li, Yongquan Wu

**Affiliations:** ^1^ Department of Cardiology Beijing Anzhen Hospital, Capital Medical University Beijing China

**Keywords:** atrial fibrillation, dual‐chamber permanent pacemaker, left bundle branch area pacing, pacing, physiological pacing

## Abstract

**Background:**

Traditional right ventricular pacing (RVP) can lead to asynchronous cardiac mechanical contractions and increase the risk of atrial fibrillation (AF). This study aimed to compare the occurrence of new‐onset AF and the progression of AF between novel physiological pacing—left bundle branch area pacing (LBBAP) and RVP with a long‐term follow‐up.

**Methods and Results:**

Patients with a dual‐chamber permanent pacemaker initial implantation, no history of persistent AF, and an expected high proportion of ventricular pacing (VP ≥ 20%) were included in this retrospective cohort study (LBBAP, *n* = 122; RVP, *n* = 166). The pacing QRS duration (QRSd) of the LBBAP was significantly shorter than that of the RVP (113 ± 22 vs. 140 ± 27 ms, *p* < 0.001), while the intrinsic QRSd values from the two groups were comparable. During a mean follow‐up of 21.9 ± 9.4 months, the composite outcome of postoperative new‐onset AF or AF progression was higher in the RVP group than in the LBBAP group (RVP HR 2.62, 95%CI 1.21–5.67, *p* = 0.014). Left ventricular end‐diastolic diameter (LVEDD) levels decreased in the LBBAP group at 1 year follow‐up (50 ± 6 vs. baseline 48 ± 6, *p* = 0.002).

**Conclusions:**

In a mean follow‐up period of 2 years, compared to RVP, LBBAP patients with VP ≥ 20% had a decreased risk of occurrence and progression of AF events.

## Graphical abstract

The retrospective cohort study aimed to compare the occurrence of new‐onset AF and AF progression between novel physiological pacing—LBBAP and RVP with nearly 2‐year follow‐up. Patients with a dual‐chamber pacemaker, no history of persistent AF, and an expected ventricular pacing (≥ 20%) were included (LBBAP, *n* = 122; RVP, *n* = 166). Compared to RVP, LBBAP patients had a decreased risk of occurrence and progression of AF events.

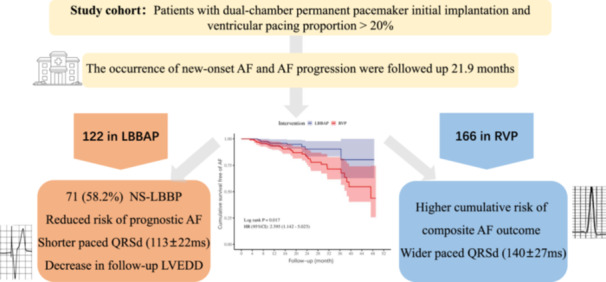

## 1 Introduction

In clinical practice, traditional pacing methods include right ventricular septum and apex pacing. The pacing sequence is opposite to that of normal cardiac electrical conduction, which can lead to electromechanical desynchrony and increase the risk of AF [1, 2]. LBBAP is a novel physiological pacing modality first introduced by Huang et al. [3] in 2017. The pacing signal depolarizes the left ventricle through the conduction system of the LBB and its branches to achieve ideal electromechanical synchronization and electrophysiological stability [4, 5]. Previous studies have confirmed the validity of LBBAP for long‐term efficacy and safety [6, 7]. Studies have also demonstrated the benefits of LBBAP in improving clinical outcomes, including mortality and rehospitalization, compared with traditional RVP [8, 9]. However, few studies have focused on the effect of LBBAP on the risk of postoperative AF in patients with a high percentage of ventricular pacing (VP%). The present study aimed to explore the effects of LBBAP on new‐onset AF and AF progression in postoperative patients compared with RVP in a long‐term follow‐up.

## 2 Materials and Methods

### 2.1 Study Design

The study was designed as a retrospective cohort analysis performed at the Beijing Anzhen Hospital Affiliated of Capital Medical University. Bradycardia patients with successful implantation of a dual‐chamber permanent pacemaker were consecutively enrolled between January 1, 2020 and January 1, 2021. This study was approved by the Institutional Review Committee, and the requirement for informed consent was waived.

### 2.2 Subject Selection

Patients aged between 18 and 90 years who had pacing indications based on the current guidelines and successfully underwent dual‐chamber permanent pacemaker implantation with postoperative VP ≥ 20% [10] and at least 1‐year follow‐up duration were enrolled in the study. Patients with clinical heart failure and left ventricular ejection fraction (LVEF) ≤ 35%, indicating implantable cardioverter‐defibrillator or cardiac resynchronization therapy, valvular heart disease (mitral or aortic valve moderate–severe regurgitation/stenosis), a history of cardiac surgery within the past 6 months, a history of persistent or long persistent atrial fibrillation, a known history of radiofrequency ablation of AF or the atrioventricular node, and those with malignancies were excluded. The study determined whether patients had a VP > 20% at the first follow‐up, which was 6 months for some patients and 3 months for others. VP percentage was determined as the mean value of the data from all device interrogations for each patient.

### 2.3 Surgical Definitions

Traditional RVP is defined as right ventricular septal pacing. The enrolled patients with LBBAP could be either nonselective or selective, and the surgery was determined to be successful if the following criteria were met, as described in the surgical record: (a) deep septal placement of the pacing lead confirmed by the fulcrum sign, (b) right bundle branch conduction delay pattern in lead V1, and (c) V6 RWPT < 74 ms or < 80 ms (patients with left bundle branch block (LBBB)). In this study, the LBBAP group used the Medtronic SelectSecure 3830 lead for VP, whereas the RVP group used Medtronic, Abbott, Biotronik, or Boston Scientific conventional active fixation leads.

### 2.4 Data Collection

The clinical data of the enrolled patients included sex, age, height, weight, arrhythmia disease such as sick sinus syndrome (SSS) and atrioventricular block (AVB), comorbidities, and New York Heart Association (NYHA) Classification of cardiac function. Paroxysmal AF (PAF) was defined as AF that terminated spontaneously or followed an intervention within 7 days of the onset of AF, with ≥ 2 episodes in the past 6 months.

The patients' baseline and 1 year follow‐up echocardiograms were conducted in accordance with the recommendations of the American Society of Echocardiography. Standard apical four‐ and two‐chamber views were selected, and the LVEDD and left atrial anteroposterior diameter (LA) were measured. LVEF was calculated using the modified single‐plane Simpson method. The patients' baseline and postoperative and 1 year follow‐up ECG parameters were collected, including the QRS duration (QRSd), baseline LBBB or right bundle branch block (RBBB). The paper travel speed for all ECGs was 100 mm/s. All QRS widths were measured in lead V1, and paced QRS width was measured from the beginning of the pace signal to the end of the QRS complex.

### 2.5 Endpoints

Follow‐up was conducted at the clinic, usually the first and third months after implantation, and then once every 6 months. AF episodes were disclosed by the pacemaker programmer at these checks. The primary endpoints were new‐onset AF and AF progression. New‐onset AF was defined as the first occurrence of paroxysmal, persistent, long‐lasting, or permanent AF after pacemaker implantation in a patient with no previous history of AF, which required a single episode of AF to be observed during a pacemaker programming onset time of > 30 s [11]. Atrial high‐rate events were recorded during pacemaker programming, which required further ECG or Holter monitoring to confirm whether they were AF. AF progression was defined as PAF before implantation and an increase of ≥ 5% [10] in the AF burden observed when programmed during regular review. The pacemaker was programmed again before the patients were discharged, and the total stay was 5 days. During this period, the pacemaker program usually showed the AF burden, and the value at this time was considered the initial value and compared with the value at follow‐up.

### 2.6 Statistical Analysis

SPSS 23.0 and R 3.4.4 software were used for statistical analysis. Patients were divided into two groups according to the surgical method: LBBAP and RVP. The clinical information of the patients was compared between the two groups. Continuous variables at baseline and at 1 year follow‐up (echocardiographic indicators and electrocardiographic indicators) were compared using paired *t*‐tests if the variables followed normal distributions and rank‐sum tests if the variables did not have normal distributions or had unknown distributions. The number of new‐onset AF events and the progression of AF events were recorded during follow‐up, and the event rate for each group was calculated. Using the Kaplan–Meier method, the survival curves of the two surgical groups and the incidence of AF events were established. The log‐rank test was used to compare the survival time differences between the curves and to clarify the effect of the type of surgery on the prognosis of AF in each group of patients. A Cox proportional hazards regression model was used to analyze the association (univariate and multivariate) between the two surgical procedures and AF events. *p* < 0.05 indicated that the difference was statistically significant.

## 3 Results

### 3.1 Baseline Clinical Characteristics

**Figure 1:**
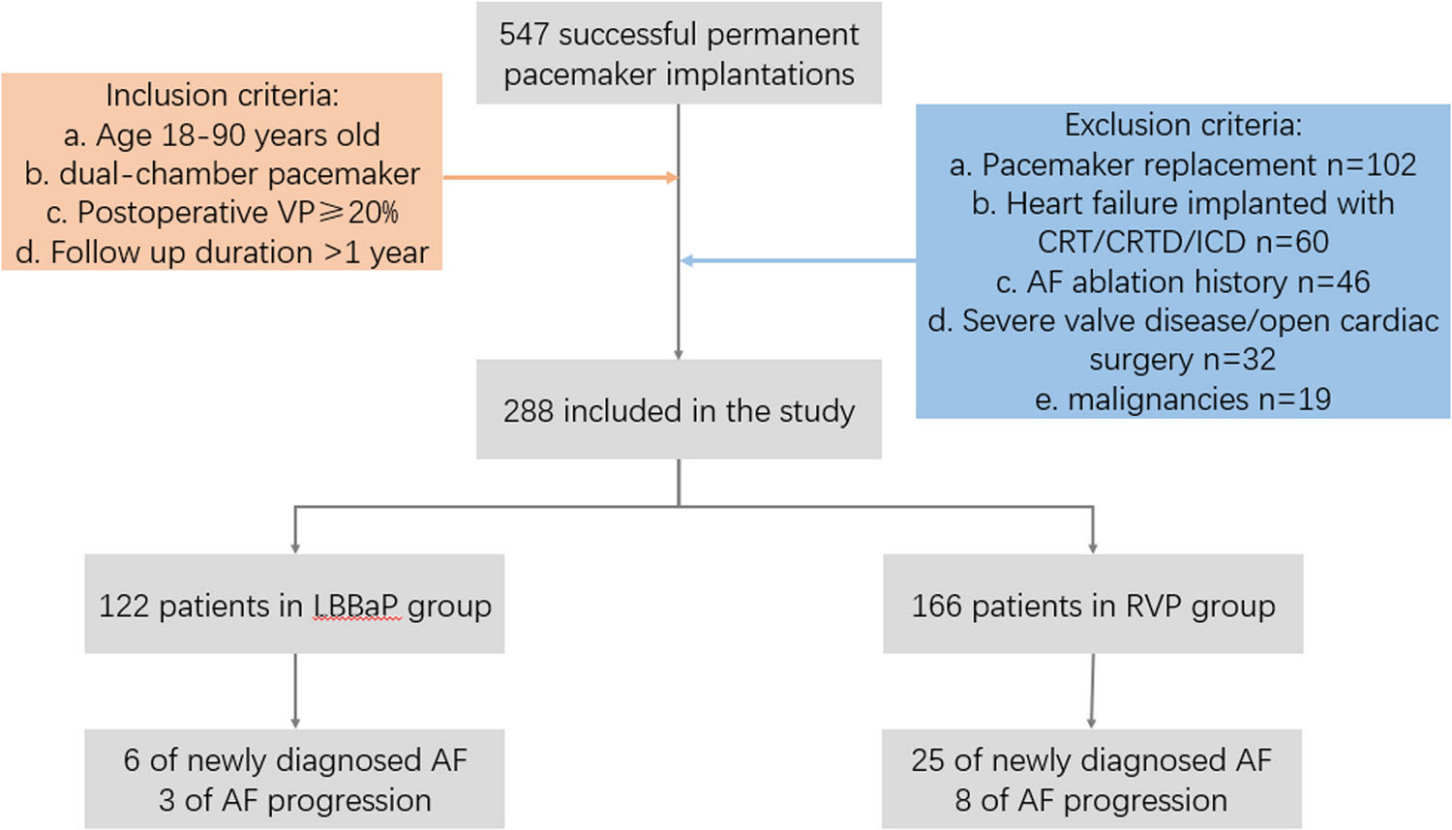
Flowchart of screened patients in the study according to the inclusion and exclusion criteria.

A total of 547 patients with symptomatic bradycardia, treated with permanent pacemaker implantation at our institution, were screened between January 2020 and December 2021. Based on the inclusion and exclusion criteria, 288 patients were included in this study (Figure 1). Patients received LBBAP or RVP therapy from seven implanting surgeons. Unless the patient had a strong requirement for the pacemaker brand or pacing modalities, the surgeon generally chose the operating methods based on their own consideration. An AV delay was set when the patient was discharged from the hospital. For 223 patients diagnosed with AVB, the pAV was 180 ms and the sAV was 150 ms. The 65 patients diagnosed with SSS were all set with AV search/AAI‐DDD if AVB was not combined. If these patients had SSS combined with AVB. The pAV was set to 180 ms and the sAV to 150 ms. The upper and lower rates were set at 130/60 in both the groups. The baseline characteristics of the study population are shown in Table 1.

**Table 1:** Clinical characteristics of patients by two pacing modalities.

Variables	Total (*n* = 288)	LBBAP (*n* = 122)	RVP (*n* = 166)	Statistic	*p*
Age, years	66.1 ± 12.4	64.5 ± 13.2	67.3 ± 11.7	*t* = −1.92	0.056
Male, *n* (%)	162 (56.25)	73 (59.84)	89 (53.61)	*χ* ^2^ = 1.11	0.293
BMI, kg/m^2^	25.1 ± 3.7	25.4 ± 3.6	24.8 ± 3.8	*t* = 1.24	0.214
SBP, mmHg	138.3 ± 19.1	138.8 ± 19.7	138.0 ± 18.7	*t* = 0.34	0.734
DBP, mmHg	72.2 ± 11.2	74.1 ± 10.8	70.7 ± 11.3	*t* = 2.57	0.011
Smoke, *n* (%)	29 (10.1)	10 (8.2)	19 (11.5)	*χ* ^2^ = 0.82	0.365
Intrinsic electrocardiography					
QRS duration, ms	104.9 ± 29.1	105.8 ± 30.8	104.2 ± 27.	*t* = 0.45	0.653
RBBB, *n* (%)	54 (18.8)	23 (18.9)	31 (18.7)	*χ* ^2^ = 0.00	0.970
LBBB, *n* (%)	38 (13.2)	18 (14.8)	20 (12.1)	*χ* ^2^ = 0.45	0.503
Baseline echocardiography					
LVEF, %	63.2 ± 6.1	62.5 ± 7.2	63.7 ± 5.1	*t* = −1.61	0.109
LVEDD, mm	48.7 ± 5.3	49.6 ± 5.5	48.0 ± 5.0	*t* = 2.46	0.015
LAD, mm	38.5 ± 5.5	38.5 ± 5.0	38.5 ± 5.9	*t* = 0.01	0.991
Pacing indications					
SSS, *n* (%)	65 (22.6)	21 (17.2)	44 (26.5)	*χ* ^2^ = 3.47	0.062
AVB, *n* (%)	223 (77.4)	101 (82.8)	122 (73.5)	*χ* ^2^ = 3.47	0.062
Follow‐up					
Duration, months	21.9 ± 9.4	21.5 ± 8.5	22.2 ± 10.0	*t* = −0.64	0.525
VP ≥ 40%, *n* (%)	257 (89.2)	112 (91.8)	145 (87.4)	*χ* ^2^= 1.45	0.228
VP ≥ 80%, *n* (%)	203 (70.5)	92 (75.4)	111 (66.9)	*χ* ^2^ = 2.47	0.116
AF history, *n* (%)	40 (13.9)	17 (13.9)	23 (13.9)	*χ* ^2^ = 0.00	0.985
Coronary artery disease, *n* (%)	55 (19.1)	26 (21.3)	29 (17.5)	—	0.236
Hypertension, *n* (%)	173 (60.1)	72 (59.0)	101 (60.9)	*χ* ^2^ = 0.10	0.754
Diabetes, *n* (%)	68 (23.6)	32 (26.2)	36 (21.7)	*χ* ^2^ = 0.80	0.370
Use of ACEI/ARB, *n* (%)	61 (21.2)	28 (23.0)	33 (19.9)	*χ* ^2^ = 0.40	0.528
NYHA, *n* (%)				—	0.099
I	244 (84.7)	97 (79.5)	147 (88.6)		
II	38 (13.2)	22 (18.0)	16 (9.6)		
III–IV	6 (2.1)	3 (2.5)	3 (1.8)		

*Note:* Data are presented as mean ± standard deviation for continuous variables and as numbers and percentages for categorical variables.

Abbreviations: ACEI, angiotensin‐converting enzyme inhibitor; AF, atrial fibrillation; ARB, angiotensin II receptor blocker; AVB, atrioventricular block; BMI, body mass index; DBP, diastolic blood pressure; LAD, left atrial diameter; LBBAP, left bundle branch area pacing; LBBB, left bundle branch block; LVEDD, left ventricular end‐diastolic dimension; LVEF, left ventricular ejection fraction; NYHA, New York Heart Association classification of functional capacity; RBBB, right bundle branch block; RVP, right ventricular pacing; SBP, systolic blood pressure; SSS, sick sinus syndrome; VP, ventricular pacing.

There were 122 patients (42.4%) in the LBBAP group and 166 in the RVP group. Of 122 patients with LBBAP, 71 (58.2%) were identified as having NS‐LBBP by pacing the QRS pattern. The mean age was 66.1 ± 12.4 years, and 162 (56.25%) were male. Both groups were treated with either LBBAP or RVP in similar proportions. The baseline LVEF was comparable between the LBBAP (63 ± 7%) and RVP (64 ± 5%) groups (*p* = 0.109). LVEDD was greater (50 ± 6 mm vs. RVP 48 ± 5 mm, *p* = 0.015), and DBP was higher (74 ± 11 mmHg vs. RVP 71 ± 11 mmHg, *p* = 0.011) in the LBBAP group. The preoperative incidence of pre‐existing PAF was 13.9%, which was equally distributed in both groups. Patients with intrinsically narrow or wide QRSds (LBBB or RBBB) were equally present in the LBBAP and RVP groups. There were no significant differences between the two groups in the remaining patient profiles or follow‐up duration.

### 3.2 Influence on the Composite Endpoints of AF

Of the 248 (86.1%) patients with no prior history of AF, 31(12.5%) were diagnosed with new‐onset AF during the mean follow‐up of 21.9 ± 9.4 months, including 7 patients with AF lasting < 6 min, 16 patients with AF lasting between 6 min and 24 h, and 8 patients with AF lasting longer than 24 h. Among them, 6 (5.7%) were in the LBBAP group and 25 (17.5%) were in the RVP group. AF progression was confirmed in 11 (27.5%) patients with previous PAF: 3 in the LBBAP group and 8 in the RVP group. Among patients with a history of PAF, there were no patients with AF lasting < 6 min, 2 patients with AF lasting < 6 min to 24 h, and 9 patients with AF lasting more than 24 h. Up to 24 patients with no history of AF had clinical symptomatic atrial fibrillation at follow‐up, including 2 in the LBBAP group and 22 in the RVP group.

**Figure 2:**
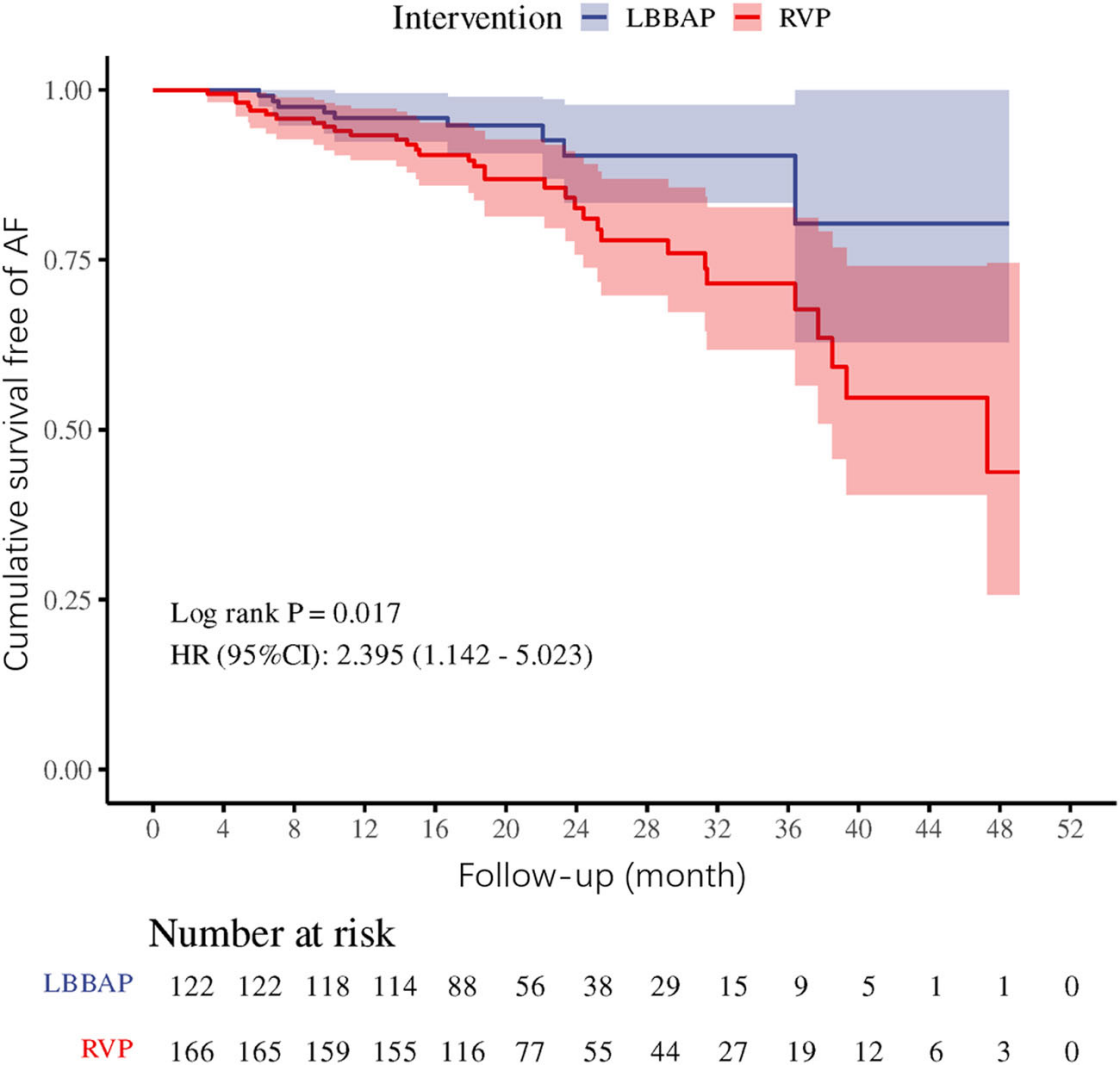
Kaplan–Meier survival curves for primary endpoint based on the groups of LBBAP and RVP.

Figure 2 illustrates the KM curves for the cumulative risk of the composite outcome of postoperative new‐onset AF or AF progression between the two groups. Compared with the LBBAP, the RVP group had a higher cumulative risk of composite AF outcome when adjusting for confounding factors [adjusted HR (95% CI): 2.395 (1.142, 5.023), log‐rank test *p* = 0.017].

As shown in Table 2, the Cox proportional hazards regression model showed a higher risk of the composite endpoint of AF in the RVP group than in the LBBAP group (HR 2.40, 95% CI 1.14–5.02). After adjusting for confounding factors such as baseline LAD and NYHA in the model, the above trend was still robust, suggesting that LBBAP had a reduced risk of prognostic AF events in this cohort (RVP HR 2.62, 95%CI 1.21–5.67, *p* = 0.014).

**Table 2:** Multivariate Cox regression analysis for risk factors of AF composite endpoint.

	HR (95% CI)		
	Model 1	Model 2	Model 3
LBBAP, Reference	1.0 (Reference)	1.0 (Reference)	1.0 (Reference)
RVP vs. LBBAP	2.40 (1.14, 5.02)	2.59 (1.22, 5.48)	2.62 (1.21, 5.67)
*p* value	0.021	0.013	0.014

*Note:* Model 1: univariate analysis; Model 2: adjusting for age, sex, hypertension, and diabetes; and Model 3: adjusting for age, sex, hypertension, diabetes, CAD, baseline LAD, baseline RBBB, NYHA, follow‐up VP%, AVB, and SSS.

### 3.3 QRS Duration and Echocardiographic Changes of 1 Year Follow‐Up

**Figure 3:**
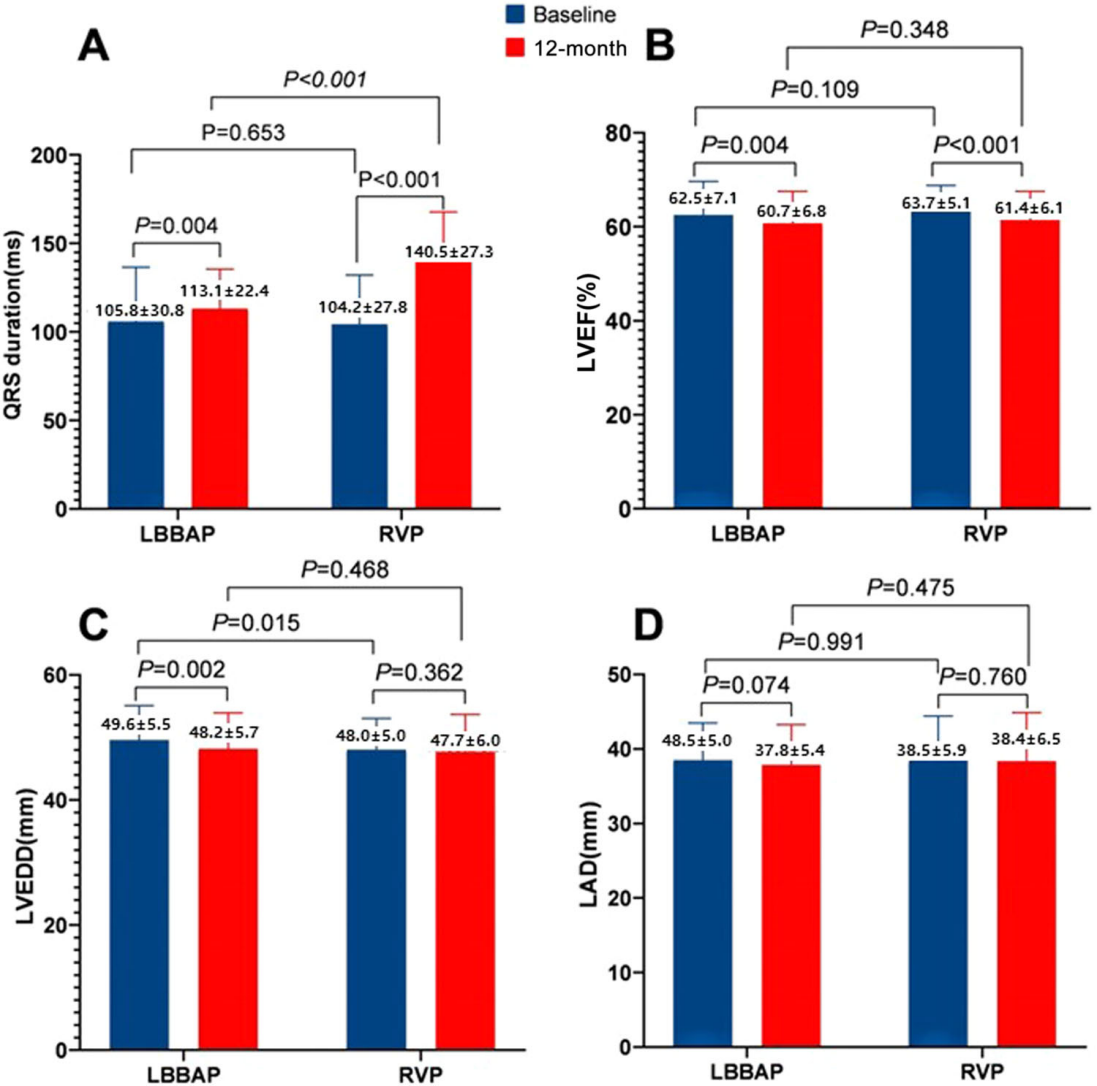
Comparison of baseline and 1 year follow‐up QRSd and echocardiography of patients with two pacing modalities. (A) Comparison of intrinsic and paced QRSd; (B–D) comparison of baseline and follow‐up echocardiography: LVEF, LVEDD, LAD.

Figure 3 shows the QRSd and echocardiographic changes in the patients over a 1‐year follow‐up. In LBBAP patients, the 1‐year bipolar paced QRSd was 113 ± 22 ms, compared with 106 ± 31 ms at baseline (*p* = 0.004). A significant increase in postsurgery QRSd was observed in with RVP patients (140 ± 27 vs. baseline 104 ± 28 ms, *p* < 0.001). One‐year paced QRS was significantly wider in RVP patients than in the LBBAP group (140 ± 27 vs. 113 ± 22 ms, *p* < 0.001), while there was no difference between the two groups in baseline QRSd (*p* = 0.653), as shown in Figure 3A.

The changes in echocardiography of the two groups at baseline and 1 year follow‐up are illustrated in Figure 3B–D. Both LBBAP and RVP patients had slightly decreased LVEF levels 1 year after implantation, while there were no differences between the two groups at admission and follow‐up (Figure 3B). Figure 3C shows that the baseline LVEDD was larger in the LBBAP patients than in the RVP patients (50 ± 6 vs. 48 ± 5 mm, *p* = 0.015) and showed a decrease in LVEDD levels in the LBBAP group at the year follow‐up (50 ± 6 vs. baseline 48 ± 6 mm, *p* = 0.002). There were no statistically significant differences in the LAD levels between the two groups at baseline or follow‐up (Figure 3D).

## 4 Discussion

In this study, patients with RVP and VP ≥ 20% had a 2.62‐fold higher risk of postoperative new‐onset AF or AF progression than patients with LBBAP after a mean follow‐up duration of up to 2 years. This suggests that the LBBAP had a reduced risk of prognostic AF events in the high‐burden RVP cohort.

In recent years, studies have found that His bundle pacing results in a lower risk of postoperative AF than RVP [10, 12]. It has also been demonstrated that LBBAP is associated with a lower incidence of sustained VT/VF and new‐onset AF than BVP [13]. Large randomized trials have provided high‐quality evidence [14] that oral anticoagulant therapy reduces stroke risk in patients with device‐detected atrial fibrillation. In the present study, the incidence of newly diagnosed AF was 5.7% in LBBAP patients and 17.5% in RVP patients at a mean follow‐up of 2 years, lower than that reported by Pastore and colleagues (HA 16.9%, RVP 25.7% and 28%) [12]. The conclusion of this study is similar to that of previous studies, suggesting that LBBAP can provide a protective effect against the occurrence and progression of postoperative AF compared with traditional RVP.

In this study, the occurrence and progression of AF in patients with a relatively high proportion of VP may be due to several reasons [15, 16, 17]. Pacing at the septum of the right ventricle causes the electrical and mechanical activation sequence of the myocardium to pass from the apex or septum to the bottom of the heart, which is the opposite of the normal activation sequence, resulting in a delay in left ventricular activation and asynchrony between the left and right ventricles. This is identified as an iatrogenic LBBB, which leads to an increase in left ventricular end‐systolic pressure and volume, resulting in an overload of the left atrial pressure and volume. Excessive traction leads to atrial fibrosis, which can cause decreased atrial wall compliance. Studies using cardiac ultrasound have confirmed that the increase in filling pressure caused by traditional RVP can lead to dysfunction of the atrial active pump [15]. All of these pathophysiological changes eventually lead to atrial electromechanical remodeling, which may promote the occurrence of AF. Therefore, ventricular asynchrony caused by nonphysiological pacing may have been the culprit of AF.

Ultrasound studies compared at baseline and at 1 year indicates a trend of cardiac structural changes in patients with pacing. Our study found that LVEDD levels decreased in the LBBAP group at 1 year follow‐up. Statistically significant differences in LVEF and LAD between the two pacing approaches were not observed in our study. This could be attributed to our relatively small sample size, which might have underpowered the study to reveal any differences in the cardiac mechanical function. In addition, most of our study cohort patients were relatively healthy, had normal cardiac function, and had few cardiovascular comorbidities. The NYHA class I–II population accounted for a high proportion in our study, which makes it less likely to cause significant changes in left ventricular systolic function.

This study had several limitations. The detection of postoperative AF using a pacemaker programmer is not absolute. Some patients may have had a history of AF before implantation; however, they were not captured during the evaluation at admission. If the AF of these patients is recorded by a pacemaker, they will be misjudged as having new‐onset AF, which can lead to a statistical bias. In addition, there were a limited number of patients with a history of PAF in this study; therefore, the endpoint of AF progression was not analyzed separately. The differences in pacemaker manufacturers and leads used in this study may also affect the outcome of AF endpoints. Since this was an observational study, we cannot determine what kind of pacing method should be selected when bradycardia patients have different expected VP proportions or a prior history of AF. Further randomized, prospective studies are required to validate these observations and guide clinical applications.

## 5 Conclusions

Compared with RVP, LBBAP patients with VP ≥ 20% had a lower risk of occurrence and progression of postoperative AF at a mean follow‐up of 2 years.

## Author Contributions

Yongquan Wu designed the study. Zefeng Wang, Jieruo Chen, and Fei Hang performed experiments. Jieruo Chen analyzed the data and wrote the manuscript. All authors contributed to the editorial changes in the manuscript. All authors have read and approved the final manuscript. All authors participated sufficiently in the work and agreed to be accountable for all aspects of the work. The authors are responsible for the accuracy and completeness of their references.

## Ethics Statement

The study was approved by the Ethics Committee of Anzhen Hospital as 2024154X.

## Consent

The requirement for informed consent was waived as used identified statistics before and after patients discharged.

## Conflicts of Interest

The authors declare no conflicts of interest.

## Data Availability

The data underlying this article will be shared upon reasonable request by the corresponding author.
